# Insights into nitrogen metabolism in the wild and cultivated lettuce as revealed by transcriptome and weighted gene co-expression network analysis

**DOI:** 10.1038/s41598-022-13954-z

**Published:** 2022-06-14

**Authors:** Pawan Kumar, Renee L. Eriksen, Ivan Simko, Ainong Shi, Beiquan Mou

**Affiliations:** 1 Crop Improvement and Protection Research Unit, USDA-ARS, 1636 E Alisal St, Salinas, CA 93905 USA; 2 Forage Seed and Cereal Research Unit, USDA-ARS, 3450 SW Campus Way, Corvallis, OR 97331 USA; 3 Department of Horticulture, University of Arkansas, Fayetteville, AR 72701 USA

**Keywords:** Plant sciences, Plant genetics

## Abstract

Large amounts of nitrogen fertilizers applied during lettuce (*Lactuca sativa* L.) production are lost due to leaching or volatilization, causing severe environmental pollution and increased costs of production. Developing lettuce varieties with high nitrogen use efficiency (NUE) is the eco-friendly solution to reduce nitrogen pollution. Hence, in-depth knowledge of nitrogen metabolism and assimilation genes and their regulation is critical for developing high NUE varieties. In this study, we performed comparative transcriptomic analysis of the cultivated lettuce (*L. sativa* L.) and its wild progenitor (*L. serriola*) under high and low nitrogen conditions. A total of 2,704 differentially expressed genes were identified. Key enriched biological processes included photosynthesis, oxidation–reduction process, chlorophyll biosynthetic process, and cell redox homeostasis. The transcription factors (TFs) belonging to the ethylene responsive factor family and basic helix-loop-helix family were among the top differentially expressed TFs. Using weighted gene co-expression network analysis we constructed nine co-expression modules. Among these, two modules were further investigated because of their significant association with total nitrogen content and photosynthetic efficiency of photosystem II. Three highly correlated clusters were identified which included hub genes for nitrogen metabolism, secondary metabolites, and carbon assimilation, and were regulated by cluster specific TFs. We found that the expression of nitrogen transportation and assimilation genes varied significantly between the two lettuce species thereby providing the opportunity of introgressing wild alleles into the cultivated germplasm for developing lettuce cultivars with more efficient use of nitrogen.

## Introduction

Nitrogen (N) is one of the most essential plant nutrients required by the plants for growth and development. N is a key constituent of nucleic acids, proteins, amino acids, many cofactors, and secondary metabolites. It also plays a pivotal role in regulation of several biological process like carbon metabolism, amino acid metabolism and protein synthesis^1^. N starvation impacts several major physiological and biological processes in plants affecting growth and development^2^, root architecture^3^, lignin content^4^, anthocyanin, phosphorus, and potassium content^5^, chlorophyll synthesis^6^, senescence, photosynthesis^7^, and CO_2_ assimilation^1^. Therefore, to increase crop yields, application of N fertilizers has dramatically increased in recent decades^2^. However, up to half of the applied N remains unused causing water eutrophication and air pollution by release of nitrous oxide thereby contributing to global climate change^8,9^. In fields, nitrate (NO_3_^-^) is the major source of N for plants which is reduced to ammonium (NH_4_^+^) ions and is incorporated into amino acids that are vital for plant growth^10^. Nitrate is also involved in signaling pathways regulating expression of genes in plant system^11,12^. Several genes control N metabolism pathways (N-uptake, N-translocation, N-assimilation and N-remobilization) in plants. Major genes involved in N metabolism are nitrate transporters (nitrate, ammonium), reductase (nitrite, nitrate), synthase (glutamine, glutamate), aminotransferase (aspartate, alanine), and glutamate dehydrogenase and the expression of these genes varies under different N regimes^13^.

Lettuce, *Lactuca sativa* L., cultivation is resource intensive; it is reliant on high amounts of N fertilizer application for its productivity and quality^14^. It is a shallow rooted crop, and therefore requires frequent irrigation which often leads to leaching of nitrate into groundwater. Over 75% of the total lettuce produced in USA comes from California, predominantly from the Salinas Valley, where the groundwater in agricultural region is adversely impacted due to nitrate leaching^15^. The amount of nitrate in a majority of wells in the Salinas Valley area exceeds the federal drinking water standard of 10 mg/L^16,17^. Therefore, it is important to determine methods to reduce N fertilizer application and improve nitrogen use efficiency (NUE) of lettuce without affecting its yield and quality.

NUE is a complex trait that involves several molecular, biochemical, and physiological processes and have two major components, N uptake efficiency (NUpE) and N utilization efficiency (NUtE)^18^. Improved NUE often leads to increase in above-ground biomass, seed production, protein content and overall economic yields^19^. Currently, research efforts are underway to improve NUE through a variety of approaches including mode and timing of fertilizer application^20^, crop rotation^21^, and management practices^22,23^. From the breeding perspective, two approaches to improve NUE may be undertaken. The first approach involves traditional breeding strategies in conjunction with quantitative trail loci (QTL) mapping and marker assisted selection. Several studies were conducted to improve NUE in lettuce using this approach. For example, genetic variation in NUpE was explored among lettuce cultivars which revealed high diversity in lettuce germplasm for root growth, resource capture and NUE^24,25^. Molecular breeding efforts to improve NUE in lettuce by selecting for robustness trait associated plants with deeper root systems and efficient resource capture resulted in identification of QTLs for below ground traits and shoot traits^26^.

The second strategy to improve NUE involves a targeted approach of identifying specific genes associated with N metabolism pathways and developing engineered plants with modified gene expression. Several genome-wide transcriptomics have been conducted to identify N metabolism genes and investigate responses to N stress in various plants such as Arabidopsis^27–29^, rice^30–33^, maize^34–36^, wheat^37,38^, brassica^10,39^, spinach^40^, potato^41^. However, the efforts to identify candidate genes associated with N metabolism and transcriptomic response of different lettuce species to N stress is lacking, though QTLs related to NUE has been recently mapped^42^.

To tackle this issue, here we performed an RNA-sequencing based comparative transcriptomic analysis of cultivated lettuce *L. sativa* cv Salinas and its wild progenitor *L. serriola* acc. US96UC23^43^ grown under high and low nitrogen conditions. *L. sativa* is a widely cultivated, high biomass producing species but is extremely susceptible to water and nutrient stress conditions. In contrast, the wild progenitor *L. serriola* L., often referred to as ‘prickly lettuce’, is more drought tolerant with an ability to survive under adverse conditions^44^ and is considered as invasive weed in several parts of Australia and the US (http://www.weedscience.org). We hypothesized that the *L. serriola* may carry genes or allele variants that enable it to survive under water and nutrient stress conditions while the *L. sativa* carry genes for efficient nutrient metabolism under favorable conditions. Therefore, the objective of this study was to identify key N metabolism-related genes in the two lettuce species under favorable and N stress conditions and further evaluate co-expressing genes modules affecting important traits such as photosynthetic efficiency of photosystem-II (PSII), chlorophyll content and cell detoxification. The genes identified in this study can be used for improving NUE of cultivated lettuce varieties either by introgression breeding or by genetic engineering.

## Results

### Phenotypic measurements of nitrogen related traits

**Figure 1:**
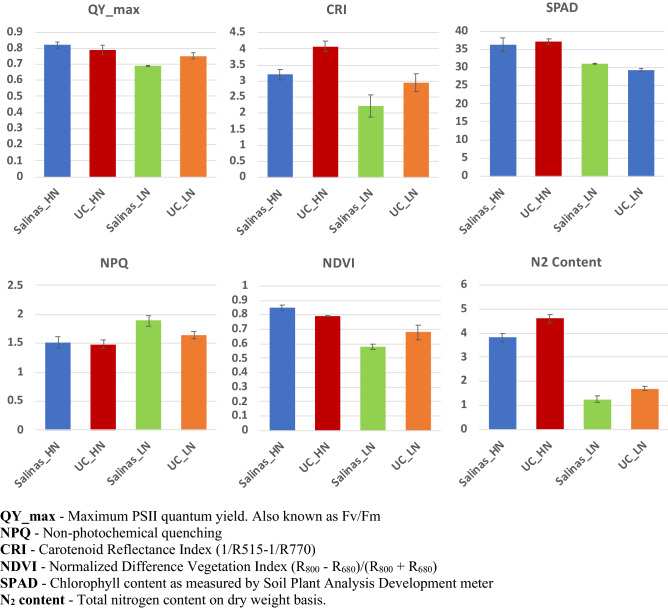
Phenotypic reaction of two lettuce genotypes to high nitrogen (HN) and low nitrogen (LN) treatments.

N supply had significant effects on chlorophyll fluorescence and vegetative indices (Fig. 1) indicating changes in the chlorophyll content, chlorophyll composition and photosynthetic efficiency due to limited availability of N. Overall, the effects of low N (LN) conditions were more prominent in Salinas than UC. Under LN conditions the total N accumulation and the photosynthetic efficiency of PSII (QY_max) was reduced by 67% and 16% respectively in Salinas compared to 63% and 5% reduction in the UC genotype. The percent reduction in SPAD was 21.3% in the UC but was significantly lower in Salinas at 14.1%. The non-photochemical quenching (NPQ) increased in both genotypes under N limited conditions (Fig. 1).

### Transcriptome sequencing and differential gene expression analysis under nitrogen stress

**Figure 2:**
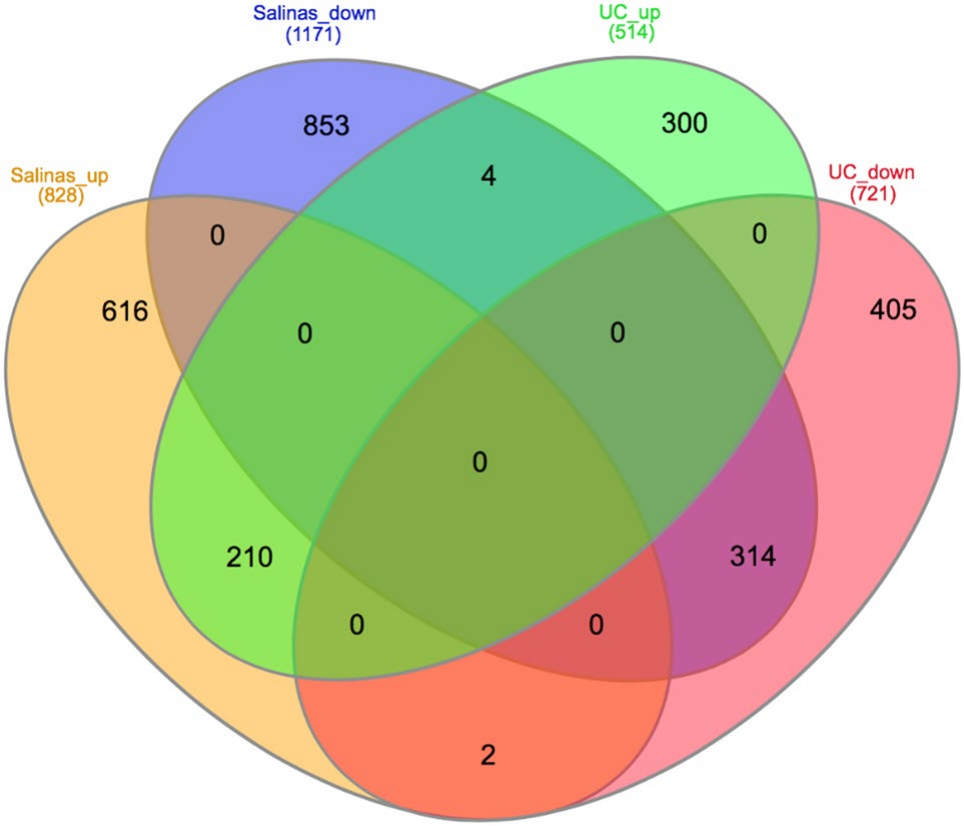
Venn diagram showing the number of up- and down regulated genes in Salinas and UC in response to nitrogen stress.

A total of 12 libraries from three biological replicates per genotype per treatment were developed. After trimming adapters and low-quality reads, a total of 300.84 million clean reads (150 bp long) were generated with an average of 25 million reads per sample. A total of 273.21 (90.81%) million reads were mapped on to the lettuce reference genome (V8) using STAR aligner software with an average of 22.76 million reads per sample (Table 1). There was a stronger response to N starvation in Salinas compared to UC. A total of 1,999 DEGs among high N (HN) and LN treatments were identified in the Salinas genotype of which 828 (41.42%) genes were upregulated and 1,171 (58.58%) genes were downregulated under LN stress condition (Fig. 2, Table S1). In UC genotypes, a total of 1,235 differentially expressed genes (DEGs) were identified of which 514 (41.62%) were upregulated while 721 (58.38%) genes were downregulated in response to N stress (Table S1). These differences in the number of DEGs suggest that the response to N stress varied between the wild and cultivated species. To validate these results, we performed RT-PCR analysis in both genotypes using key genes involved in N metabolism (Table S2). Results from RT-PCR show that the expression trends of the selected genes were in accordance with the expression detected by the RNA-seq analysis.

**Table 1:** Summary of RNA-Seq performed on two lettuce genotypes under high nitrogen (HN) and low nitrogen (LN) treatments.

Genotype	Treatment	Replicate	Library name	Total clean Reads	Mapped reads	Mapped reads (%)	Multiple Loci reads	Multiple Loci reads (%)	Unmapped reads	Unmapped reads (%)
Salinas	HN	R1	Salinas_HN1	25,413,058	22,885,448	90.05	1,056,980	4.16	1,470,630	5.79
Salinas	HN	R2	Salinas_HN2	26,614,697	23,869,149	89.68	1,284,629	4.83	1,460,919	5.49
Salinas	HN	R3	Salinas_HN3	27,246,323	24,925,925	91.48	1,123,729	4.12	1,196,669	4.39
US96UC23	HN	R1	UC_HN1	24,527,750	22,232,436	90.64	934,275	3.81	1,361,039	5.55
US96UC23	HN	R2	UC_HN2	21,366,114	19,456,364	91.06	728,930	3.41	1,180,820	5.53
US96UC23	HN	R3	UC_HN3	27,134,208	24,689,908	90.99	1,012,409	3.73	1,431,891	5.28
Salinas	LN	R1	Salinas_LN1	28,084,512	25,711,442	91.55	822,793	2.93	1,550,277	5.52
Salinas	LN	R2	Salinas_LN2	21,345,239	19,213,005	90.01	826,202	3.87	1,306,032	6.12
Salinas	LN	R3	Salinas_LN3	21,576,629	19,867,359	92.08	556,433	2.58	1,152,837	5.34
US96UC23	LN	R1	UC_LN1	27,719,243	25,049,883	90.37	941,785	3.4	1,727,575	6.23
US96UC23	LN	R2	UC_LN2	23,762,434	21,834,284	91.89	763,931	3.21	1,164,219	4.9
US96UC23	LN	R3	UC_LN3	26,052,847	23,469,949	90.09	907,243	3.48	1,675,655	6.43

### Functional classification of N-deficiency-responsive genes

**Figure 3:**
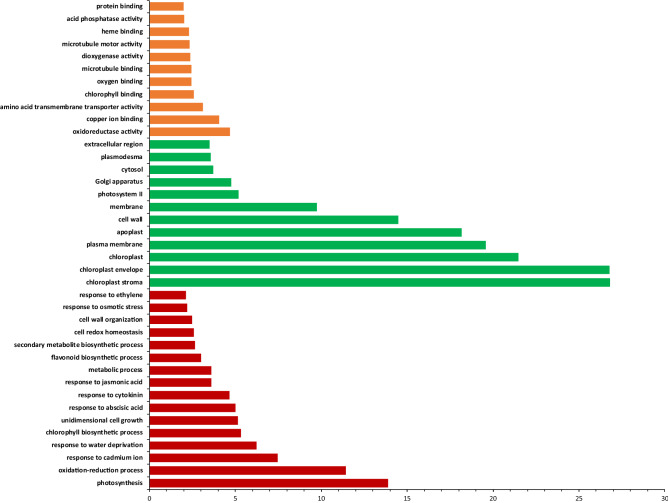
Enriched GO terms according to biological processes (red bars), cellular components (green bars) and molecular functions (orange bars). The *x*-axis represents log2 of the gene counts.

GO enrichment analysis of the annotated DEGs revealed several enriched biological processes, molecular functions and cellular components (Fig. 3). The key enriched biological processes include photosynthesis (GO:0,015,979), oxidation–reduction process (GO:0,055,114), response to water deprivation (GO:0,009,414), chlorophyll biosynthetic process (GO:0,015,995), response to cytokinin (GO:0,009,735) and cell redox homeostasis (GO:0,045,454). The key molecular functions enriched included oxidoreductase activity (GO:0,016,491), amino acid transmembrane transporter activity (GO:0,015,171), chlorophyll binding (GO:0,016,168), and protein binding (GO:0,005,515). The enriched cellular components included chloroplast stroma (GO:0,009,570), chloroplast envelope (GO:0,009,941), chloroplast (GO:0,009,507), plasma membrane (GO:0,005,886), and cytosol (GO:0,005,829). Some of the key KEGG pathways enriched are metabolic pathways (ath01100), N metabolism (ath00910), carbon metabolism (ath01200) and photosynthesis (ath00195). These results indicate that genes in these pathways play important role in host plant response to N stress.

### Differentially expressing transcription factors under nitrogen stress

**Figure 4:**
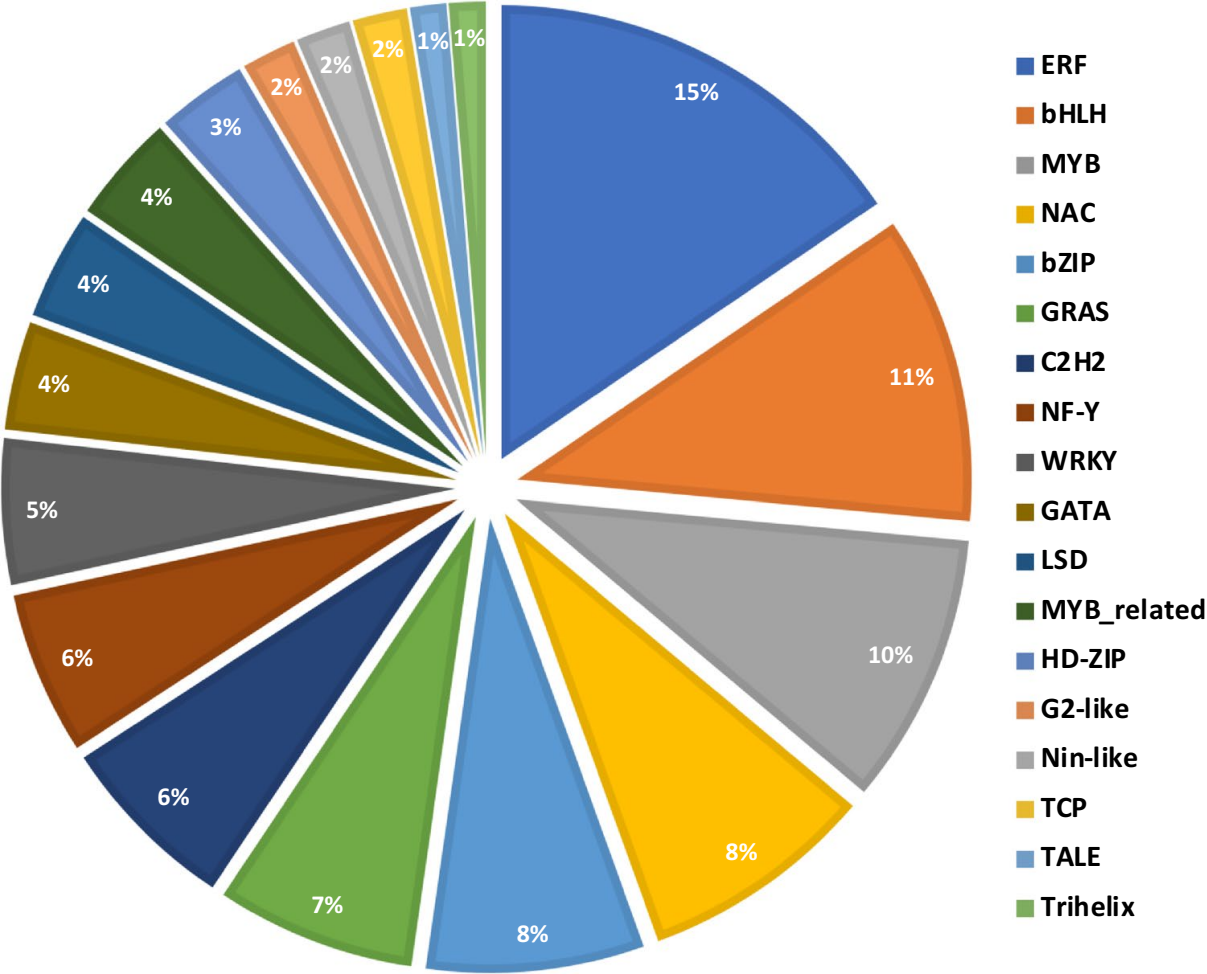
Differentially expressed transcription factors identified by RNA-Seq analysis.

Transcription factors (TFs) play key roles in plant adaptation to N stress by regulating plant signal transduction pathways. We identified total of 155 transcription factors (TFs) related genes that were differentially expressed in response to N stress. The most differentially expressed TFs (Fig. 4) belong to ethylene responsive factor (ERF) family with 24 (15%) DEGs followed by the TFs belonging to bHLH family with 17 (11%) DEGs. Other key TFs identified in this study are MYB (10%), bZIP (8%), and NAC (8%) (Fig. 3). The differential expression of TF under N stress indicate different mechanisms in the two genotypes under N stress.

### Photosynthesis and carbon assimilation related responses

**Figure 5:**
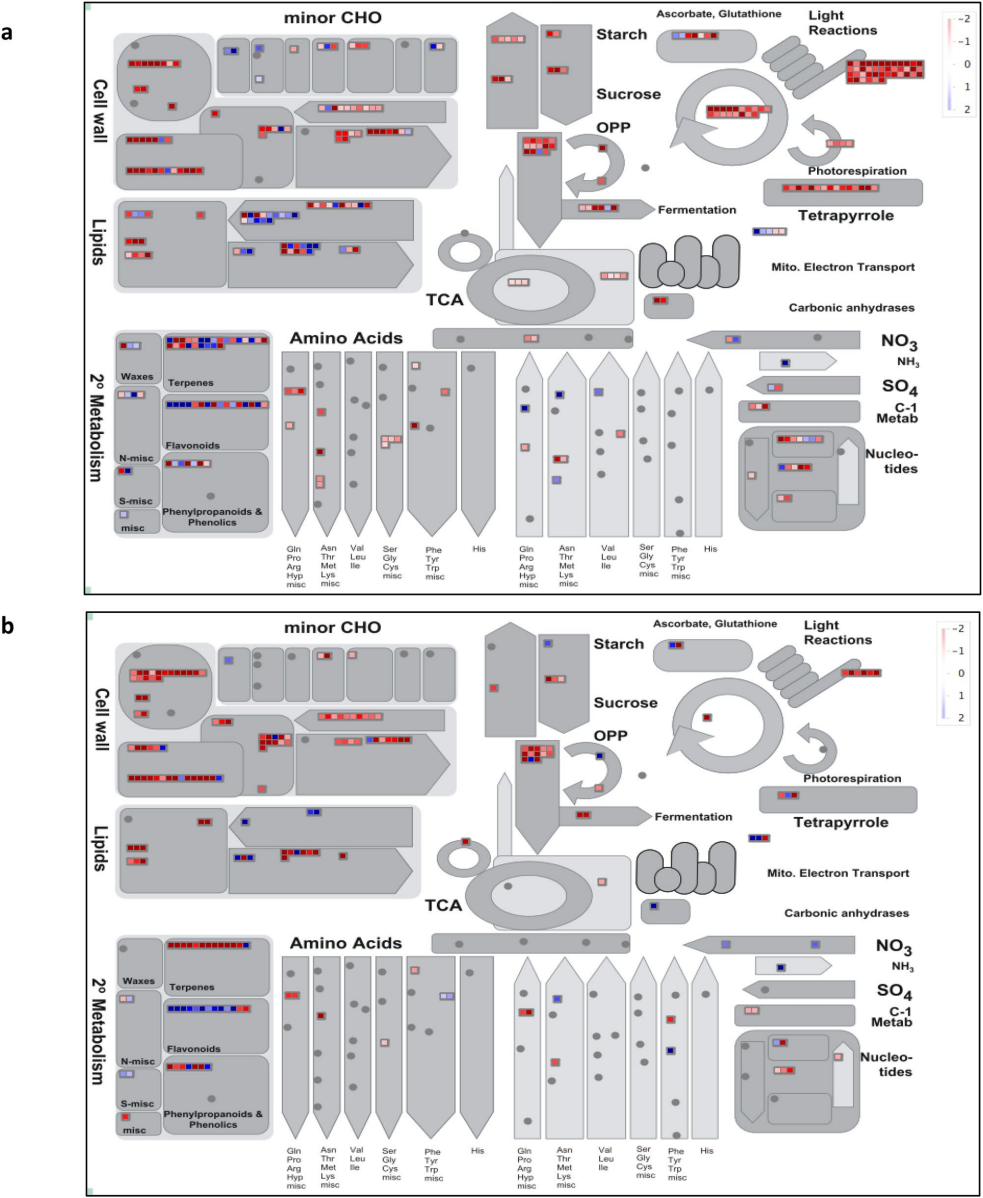
Mapman analysis of differentially expressed genes related to various metabolic processes in (**a**) Salinas, (**b**) UC genotypes. Red squares represent genes that were significantly down-regulated; blue squares represent genes that were significantly up-regulated.

Photosynthetic efficiency of Salinas and UC was reduced due to N stress. The expression of 42 photosynthesis-related genes was down regulated in Salinas which included genes encoding components of the photosystem-II light harvesting (LHC) complex, photosystem-I reaction center (PSAE, PSAF) and electron carriers (DRT112, NDF4) (Fig. 5). Major photorespiration genes (*Ls0_14580.1, Ls3_16080.1, Ls5_136860.1, Ls7_16480.1*) and 18 genes related to the Calvin cycle were also repressed under N stress including 9 RuBisCO genes encoding for ribulose bisphosphate carboxylase small chain 2B family protein. Similarly, the relative expression of photosynthesis genes in UC were also repressed, however the number of genes affected by N stress were fewer compared to Salinas. The expression levels of five genes (*Ls5_107040.1, Ls9_104661.1, Ls9_104700.1, Ls9_104780.3, Ls9_89920.1*) encoding for photosystem-II light harvesting (LHC) complex, a gene (*Ls1_35241.1*) encoding for photosystem-I reaction center and a RuBisCO gene *(Ls4_31161.1*) was downregulated in response to nitrogen stress in the UC genotype. These results indicate that the UC genotypes is able to maintain photosynthesis under N stress compared to the Salinas genotype.

### Cell wall associated responses

Effect of N stress on cell wall components was analyzed by evaluating a change in the relative expression of the cell wall precursor-related genes. Expression of 21 genes involved in biosynthesis of cell wall precursors was affected by N stress in Salinas genotype. Expression levels of only three genes (*Ls8_38141.1, Ls3_10960.1, Ls4_96981.1*) were upregulated while the expression of the remaining 18 genes was downregulated. In the UC genotype, expression of 22 cell wall precursor-related genes was affected. The relative expression of only one gene (*Ls7_110780.6*) involved in plastidial glycolytic pathway was upregulated in response to N stress (Fig. 5). We detected the expression of 13 cell wall precursor’s related genes involved in the glycolysis process, were downregulated in both genotypes. Expression of inositol oxygenase 1 (MIOX1) [Q8L799] gene (*Ls4_96981.1*) was upregulated only in Salinas genotype. This gene is involved in the biosynthesis of UDP-glucuronic acid (UDP-GlcA) thereby providing nucleotide sugars for cell-wall polymers may be crucial for cell wall associated changes under N stress.

### Secondary metabolism under nitrogen stress

Based on transcriptome analysis, response to secondary metabolite production under N stress was found to be different between the two genotypes. In Salinas, 48 secondary metabolism genes showed differential expression including 25 genes for terpenes biosynthesis, 16 genes coding for flavonoids, and 7 phenylpropanoids and phenolics biosynthesis genes (Fig. 5). Among the terpenes biosynthesis genes, the genes (*Ls5_42681.1, Ls5_100341.2*) involved in sesquiterpene (C15) biosynthesis and synthesis of tocopherol (vitamin E) that protect thylakoid membrane lipids from photooxidation were significantly downregulated (LFC > − 6.0) in Salinas genotype while the gene (*Ls1_127360.1*) involved in the biosynthesis of homoterpenes and a gene (*Ls6_52200.1*) involved in phytoene biosynthesis pathway, which is part of carotenoid biosynthesis, were significantly upregulated (LFC > 3.0) under N stress in Salinas genotype. Among the flavonoid genes, the expression of two detoxifying genes (*Ls5_149321.1, Ls9_61600.2*) involved in anthocyanin and protoanthocyanidin biosynthesis was significantly upregulated (LFC > 3.5) while the genes involved in phenylpropanoids and phenolics biosynthesis such as the Four-Coumarate:CoA ligase (4CL) encoding lettuce gene (*Ls1_18940.1*) and a gene (*Ls4_118261.1*) involved in the oxylipin biosynthetic process was the most significantly downregulated (LFC > − 7.0) gene in Salinas genotype.

Similarly, in the UC genotype, 13 genes each for terpenes biosynthesis and flavonoids and seven genes involved in phenolics biosynthesis were differentially expressing in response to N limitation. The terpenes gene (*Ls5_71000.1*) involved in lignin degradation and detoxification of lignin-derived products was significantly downregulated (LFC > − 4.0) while the cyto-detoxifying gene (*Ls9_61600.*2) was the most significantly upregulated (LFC > 5.0) gene.

### Variation in nitrogen assimilation-related genes in response to N stress

**Figure 6:**
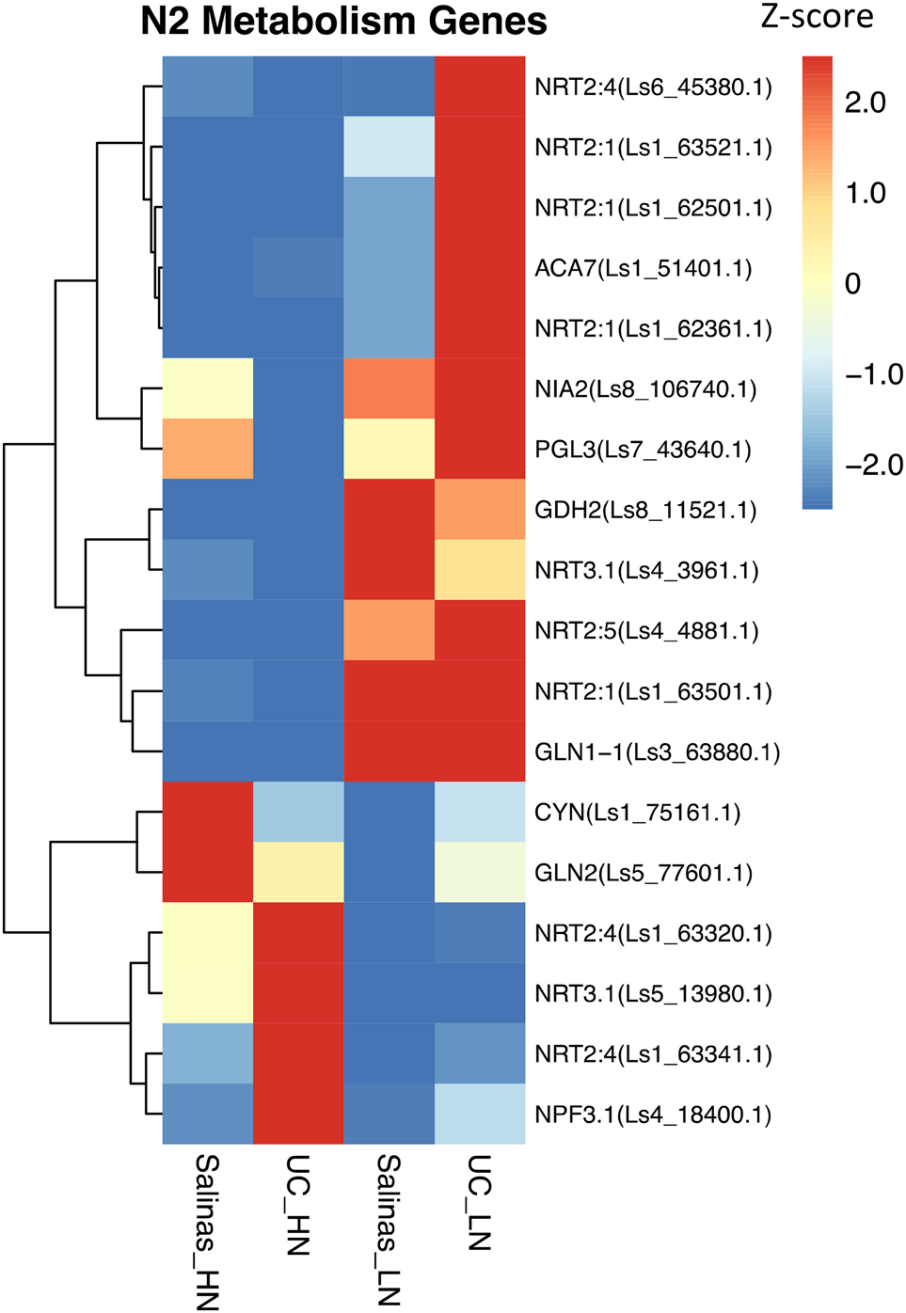
Differential expression of nitrogen metabolism genes Salinas and UC genotypes when grown at high nitrogen (HN) and low nitrogen (LN) treatments.

The expression of 12 N assimilation genes were upregulated in both genotypes while the expression of six genes was repressed under N stress. There were seven genes with higher expression in UC. Expression of genes encoding nitrate transmembrane transporters (*NRT3.1*; *Ls4_3961.1*)) and glutamate dehydrogenase 2 (*GDH2*; *Ls8_11521.1*) was higher in the Salinas genotype compared to the UC genotype. Under high N conditions, the expression of cyanase (CYN; *Ls1_75161.*1) and glutamine synthetase 2 (GLN2; *Ls5_77601.1*) genes was greater in the Salinas parent while the expression of four genes involved in nitrate uptake and transportation displayed higher expression in UC genotype under high N conditions suggesting that UC is more efficient in nitrate uptake and transfer from stored pools to cytoplasm (Fig. 6).

### Gene co-expression and network analysis

**Figure 7:**
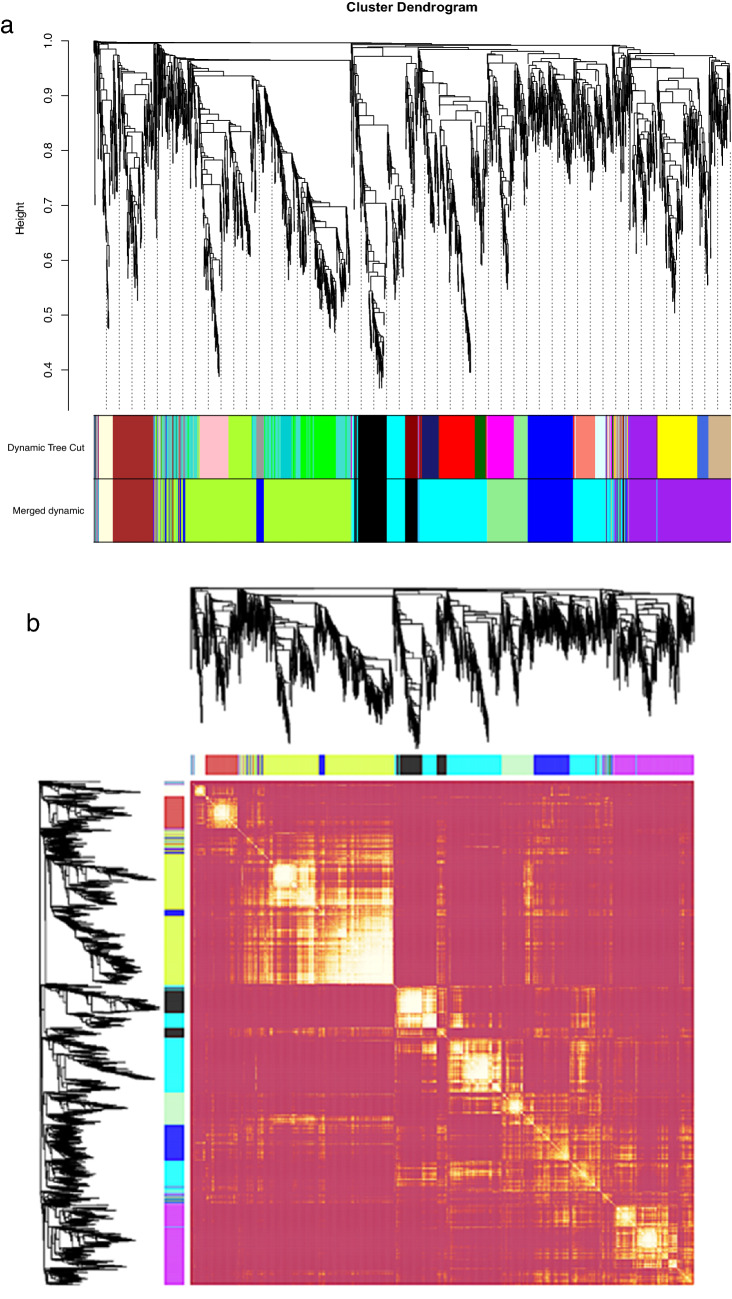
(**a**) Gene modules identified by WGCNA. Dendrogram was obtained by clustering dissimilarity with the modules represented by the colored block based on the topological overlap. A total of nine module (colored blocks) were identified where each module represents a set of highly connected genes. (**b**) Heatmap plot of the gene network. The heatmap depicts Topological Overlap Matrix (TOM) among all genes in the analysis. Light color represents overlap of highly expressed genes and the darker color represent low overlap of genes. Blocks of yellow colors along the diagonal are the modules.

**Figure 8:**
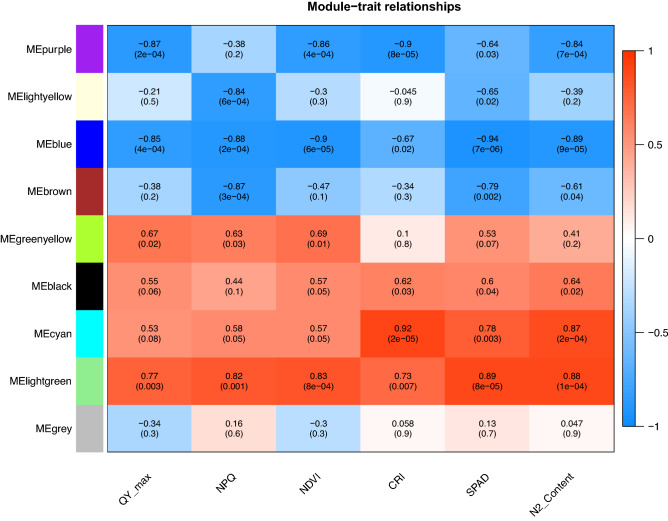
Heat map of relationship between modules and phenotypic traits. The color indicated direction of correlation, values represent Pearson correlation coefficients, and *P-values* are in parenthesis.

To get further insight into the N stress-related genes regulatory network of lettuce we used WGCNA to identify co-expression modules using expression profile of all genes having significant differential expression in Salinas and UC genotypes. The dissimilarity of the modules was set as 0.2, and 9 co-expression modules were generated by DisTOM based hierarchical clustering post dynamic tree cutting method (Fig. 7a, b). Weighted Pearson correlation coefficients calculated between eigengenes (modules) and traits (phenotypic measurements) are presented in the Fig. 8 as the module trait relationship. The light-green module was positively correlated with QY_max, NPQ, NDVI, SPAD and total N content with correlation coefficient ranging from 0.73 to 0.89. The blue module was negatively correlated with all traits with correlation coefficient ranging from − 0.67 for CRI to -0.94 for chlorophyll content (SPAD). High correlation between the module membership (MM) and gene significance (GS) for total N content was observed in light-green module (*r* = 0.7, *p-value* = 3.8e^−29^) and in the blue module (*r* = 0.68, *p-value* = 1.4e^−37^) suggesting that these modules are suitable for identifying hub genes associated with N stress associated response in lettuce (Fig. S2). The eigengene adjacency heatmap shows that N content was more adjacent to the light-green module than the blue module (Fig. S3).

**Figure 9:**
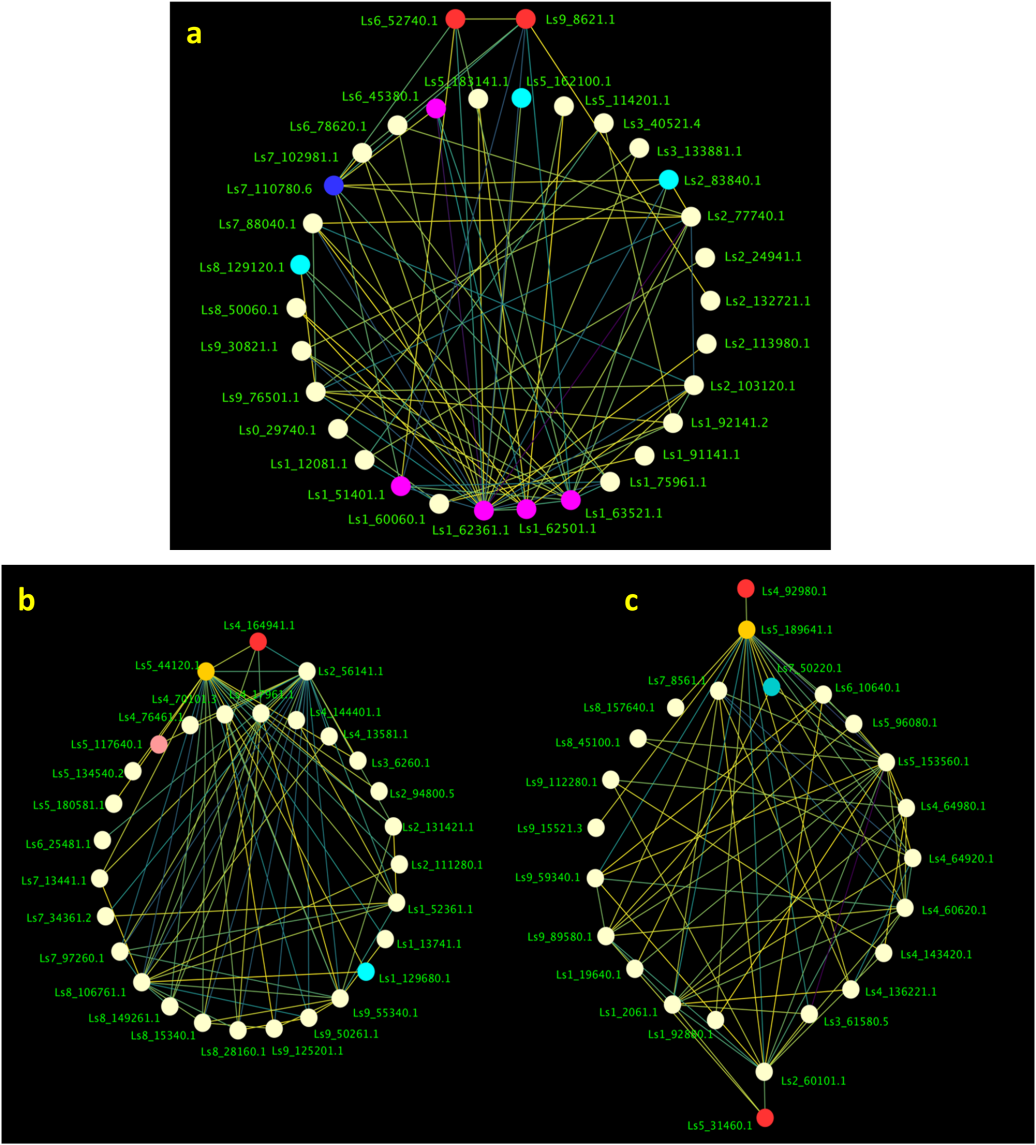
The co-expression network of highly correlated (*r*^2^ > .90) hub genes and associated TFs identified by applying the Molecular Complex Detection (MCODE) to the networks. (**a**) The co-expression network of the significant genes in the lightgreen module. (**b**, **c**). Two co-expression clusters of the significant genes in the blue module. The TFs in each cluster are red colored.

Significantly correlated edges in the light-green and blue modules were further filtered by condition of weight value being greater than 0.25. A total of 424 edges and 95 nodes were identified in the light-green module while in the blue module a total of 64 edges and 48 nodes were detected. The most significant hub clusters were identified by applying the Molecular Complex Detection (MCODE) to the networks in the light-green and blue modules (Fig. 9). A single hub cluster of highly correlated gene was identified in the light-green module (Fig. 9a). This cluster contains 32 genes including 2 transcription factors (*Ls6_52740.1, Ls9_8621.1*), 4 nitrogen metabolism-related genes (*Ls1_51401.1, Ls1_62361.1, Ls_62501, Ls1_63521.1, Ls6_45380.1*), 3 secondary metabolite genes (*Ls2_83840.1, Ls5_162100.1, Ls8_129120.1*) and a carbon assimilation gene (*Ls7_110780.6*). Two hub clusters were identified in the blue module which included 29 and 23 genes respectively (Fig. 9b, c). The first cluster appeared to be regulated by a single bHLH transcription factor (*Ls4_164941.1*) while the second cluster appeared to be regulated by a GRAS family TF (*Ls4_92980.1*) and Ethylene-responsive transcription factor RAP2-11 (*Ls5_31460.1*).

## Discussion

Transcriptome profiling using RNA-Seq enabled comparing transcriptional response of two lettuce genotypes, Salinas and UC, grown in two contrasting N regimes. The results demonstrate the common and unique differential response of these genotypes. Although some stress responsive pathways were similar in both genotypes there were significant differences at transcriptional levels in the cultivated (Salinas) and the wild (UC) lettuce to the N stress conditions.

We found that N stress adversely affected several photosynthesis related genes in lettuce such as those encoding for the light harvesting complex (LHC), the photosystem-I (PS-I) reaction center and RuBisCO. The LHC is associated with photosystem I and II which is made up of chlorophyll-a, chlorophyll-b, and binding proteins that act as light receptor to capture and deliver the excitation energy between PS-I and PS-II and also regulate the distribution of this energy under changing light conditions^45^. Similar results were reported in bread wheat^46^ and in rice^47^ where N stress significantly decreased expression of components of LHCs of both PSII and PSI. Earlier studies have documented the effect of low N on physiological processes including low photosynthetic CO_2_ assimilation and decreased photosynthetic rate^48–50^, and also reduced N^51^ and phosphorus^5^ content in leaves. In addition, insufficient N can affect photosynthetic pigments^52^, activity of Rubisco proteins^50,53^, and chlorophyll fluorescence^50^.

The photosynthetic response of the plant under abiotic stress can be quantified using chlorophyll fluorescence measurements. Our results showed that the photosynthetic efficiency (QY_max) and photosynthetic pigment concentration (SPAD values) were significantly lower in both genotypes under N stress conditions due to downregulation of genes involved in chlorophyll biosynthesis. Concomitantly, we observed a significant increase in non-photochemical quenching (NPQ) under N stress (Fig. 1). Thermal dissipation measured as NPQ, is a photoprotective mechanism that can eliminate excess irradiated energy absorbed by the plant. When plants absorb more light energy than they can utilize during abiotic stress, excess energy leads to the production of reactive oxygen species (ROS) which can cause severe damage to the plant’s photosynthetic apparatus and at higher levels can cause cell death. NPQ has been shown to increase under stresses conditions and plays an important role in the reduction in electron transport, increases in heat dissipation, and resistance to abiotic stresses^54^. Increases in NPQ in Salinas and UC genotypes suggest that dissipating the excess of excitation energy absorbed by PSII as heat is the principal pathway to reduce ROS formation under N stress conditions.

N uptake, assimilation and metabolism and its effect on growth and biomass production have been studied in several agriculturally important plant^19,55^. The process of NO_3_¯ uptake, translocation and storage in plant is a complex process and is often controlled by low-affinity transporter (LAT) and high-affinity transporter (HAT) genes^13^. Most *nitrate transporter 1* genes act as nitrate sensors and function as low-affinity transporter genes for NO_3_^−^ at high concentrations, with the exception of AtNRT1.1 in Arabidopsis and MtNRT1.1 in *Medicago truncatula* that serve as dual-affinity transporter involved in LAT and HAT systems^56^. Previous studies demonstrated upregulation of NRT1.1 upon addition of nitrate to N starved plants. For example, cultivar specific differences were observed in *Brassica juncea* for the expression of NRT1.1 and NRT1.8 that were highly induced as early as 20 min after exogenous supply of nitrate^10^. In this study we did not find significant differences for Lettuce NRT1.1 gene expression under HN or LN condition confirming the role of NRT1.1 gene in nitrate perception. In contrast to our finding^46^, found that in wheat the expression of most of the dual affinity nitrate transporters (like NRT1.1) decreased under nitrogen stress and suggested that the reduced expression of NRT1.1 genes may lead to retarded growth, low grain protein content and low grain yield of N stressed plants. We observed that the expression of many high affinity nitrate transporter NRT2 genes was altered under low N conditions. The NRT2 genes family belong to HAT system and are responsible for transporting nitrate at low concentrations, therefore they play an important role during nitrogen stress conditions^13,57,58^. Many NRT2 family members require NAR2 (nitrate assimilation related protein) for nitrate transportation and have different spatio-temporal distribution in roots^57,59,60^. In Arabidopsis, NRT2.1 mediates apoplastic nitrate absorption and affects root system architecture under low nitrogen conditions^58,61^. Unlike NRT2.1, NRT2.4 and NRT2.5 are expressed in epidermal cells of roots under long term nitrogen starvation and responsible for nitrate uptake from soil into the plant^57,60^. Compared to Salians genotype, we found induced expression of several high affinity nitrate transporter genes (NRT2.1, NRT2.4 and NRT2.5) in UC genotype under low N condition which may explain higher N content observed in UC than Salinas genotype thus indicating that UC genotype could have higher N uptake efficiency under low N conditions.

The process of converting inorganic nitrogen from soil into organic N is carried out by two key enzymes, glutamine synthase (GS) and glutamate synthase (GOGAT). GS catalyzes adenosine triphosphate (ATP)-dependent fixation of ammonium to form glutamine while the GOGAT enzyme catalyzes the conversion of glutamine to two molecules of glutamate^62^. Glutamine serves as a main nitrogen donor for biosynthesis of amino acids, nucleotides, and chlorophyll. Therefore the GS enzyme is the major factor affecting nitrogen assimilation in plants^63^. There are two isoenzyme forms of the GS enzyme, one located in the cystosol (GS1 or GLN1) and the other in chloroplast/plastids (GS2 or GLN2). The cytosolic GLN1 plays an important role in N assimilation in roots while the chloroplastic GLN2 is expressed primarily in leaves and is responsible for the reassimilation of the ammonia generated by photorespiration^63^. In this study, we found that the expression of gene for GLN1 was up-regulated under N stress in both genotypes while the expression of GLN2 was higher in Salinas under high N but was down-regulated under N stress. There was no change in the expression levels of the GLN2 in UC under high or low N conditions. Together, these results suggest that the UC can sustain N assimilation under low N condition and may account for higher N content and biomass produced during nitrogen stress.

TFs recognize specific motifs and function as a switch that can turn on or off a particular gene. In this study we found that the expression of TFs like bHLH, bZIP, ERF, MYB, NAC, and WRKY was altered in response to N stress (Supplementary_Fig. 4). The Apetala2/ethylene responsive factors (AP2/ERF) family TFs are the key regulators of numerous abiotic stresses and respond to several plant hormones^64,65^. These TFs are induced upon specific stresses and have diverse DNA binding preferences, enabling these TFs to integrate responses of multiple stimuli and participate in regulatory processes^66^. Here we found that of the 14 ERF transcription factor showing differential expression, 12 were upregulated in both, Salinas and UC under low N condition suggesting ERF TFs play a crucial role in regulating N responsive genes in lettuce. Similar results were observed in cucumber by^12^, where they identified over seven differentially expressed ERF TFs in response to early nitrogen deficiency. The basic leucine zipper (bZIP) TF family is one of the largest TF family in plants that is involved in regulating plant growth, development, and biotic and abiotic stress responses^67^. In this study, the majority of the bZIP TFs were identified in the Salinas genotype with both, up- and down-regulation of their expression in response to N stress. Functional characterization of a bZIP TF (*TabZIP*) in wheat revealed it regulated several biological processes including cellular nitrogen compound metabolic process (GO:0,034,641)^68^. In Arabidopsis, a bZIP TF *AtTGA4* was found to be induced under drought and low N stress and its over-expression resulted in improved drought tolerance and reduced nitrogen starvation^69^. The transgenic plant overexpressing *AtTGA4* had higher N and proline content than the wild type controls. The activity of N transportation (NRT2.1, NRT2.2) genes and nitrogen assimilation (NIA1, NIA2) genes were also higher in the transgenic plants. We observed that the expression of NIA2 gene (*Ls8_106740.1*) was significantly higher in the UC genotype under N stress and may be under bZIP TF regulation. The number of downregulated basic helix-loop-helix domain (bHLH) TFs were greater in Salinas than the UC genotype. Plant bHLH proteins control response to light and interact with components of the circadian clock^70^. In Arabidopsis *AtbHLH008* can regulate circadian clock genes *CCA1* and *LHY* thus providing entry point to phytochrome regulation of the circadian clock^71^. Downregulation of bHLH genes in Salinas under stress maybe a mechanism to minimize photoreception during reduced photosynthesis thereby protecting plant from ROS production. The NAC TFs control several biochemical and molecular pathways that help plants to survive under different stress conditions. In this study we found that the differentially expressing NAC TFs were upregulated in both genotypes and none were identified to be downregulated (Fig. S4) suggesting critical role of NAC TFs in N stress management in lettuce^72^ identified a NAC TF *TaNAC2-52* in wheat that could bind to the promoter regions of the genes encoding nitrate transporter and glutamine synthase. Overexpression of *TaNAC2-52* TF caused enhanced root growth with higher nitrate influx rate, resulting in higher N accumulation in aerial parts of the transgenic wheat plants. Similar results were reported for *TaNAC-S* TF where its over expression resulted in delayed leaf senescence (stay green phenotype) and higher N/protein content in grains of the transgenic wheat plants^73^.

Genes with similar pattern of expression are most likely to be under similar regulation^74^. In this study, we used WGCNA to construct gene co-expression networks and to identify hub gene. All DEGs were divided into nine modules, each of them significantly correlated with one or more phenotypic traits. Co-expression network construction highlighted several hub genes that are expected to play important roles during nitrogen stress in lettuce. All the correlated networks identified in this study were regulated by one or more transcription factors. Transcriptional regulation of N use efficiency genes is reported in tea^75^, soybean^76^, Arabidopsis^77^, brassica^10^, bean^78^. The carbon assimilation gene and high affinity nitrate transporter genes were identified in the lightgreen module cluster regulated by ERTFs (RAP2-11, ERF098) that are associated with the biological function of response to ROS and are involved in regulation of gene expression by stress factor and by components of stress signal transduction. Regulation of the nitrate transporter genes by RAP2-11 was demonstrated in Arabidopsis^79^. Similar results were also observed in cucumber where the ERF TFs regulated photosynthesis related genes, nitrogen deficiency responsive genes and carbon assimilation genes^12^.

A major hub gene *Ls5_44120.1* encoding for PLAT domain-containing protein 2 (PLAT2) appear to be interacting with large number of genes in the blue module cluster. In Arabidopsis the PLAT 1 gene is shown to be regulated by bZIP TF and is critically involved in abiotic stress tolerance^80^. Two hub genes, *Ls5_189641.1* and *Ls2_60101.1* interacted with many genes in the second cluster of the blue module. The gene *Ls5_189641.1* encoding for Glutaredoxin-C13 (GRXC13) is associated with cellular response to N starvation and has been shown to mediate signaling and plant response to nitrate starvation in Arabidopsis^81^ while the gene *Ls2_60101.1* for cationic amino acid transporter 5 protein (CAT5) is involved in transport of the amino acids. The CAT genes are part of amino acid transporters (AATs), are protein that perform inter- and intra-cellular movements of amino acids after nitrogen assimilation therefore are important for nitrogen homeostasis in plants^82–84^. Altered expression of these hub genes may account for the positive and negative correlations of the light-green and blue modules with the phenotypic traits including the photosynthetic efficiency of PSII, SPAD, and total nitrogen content in lettuce.

## Conclusion

In this study, the comparative transcriptomic analysis of the cultivated and wild lettuce provided us valuable insight into the differential transcriptional responses of the two lettuce species to N starvation. A large number of genes were identified that showed up- or down-regulation under N stress.

The expression data provided information on the effects of N stress on genes that control important physiological processes such as photosynthesis, carbon assimilation, cell wall formation and glycolysis. This study also provided insight into species-specific N metabolism and assimilation genes which may explain the phenotypic variation observed for the N related traits. In addition, the co-expression network analysis of the 2704 differentially expressed genes obtained from the RNA-seq data of the two lettuce species revealed key hub genes and regulatory modules that might be associated with the regulation of carbon–nitrogen interactions and secondary metabolite biosynthesis. The focus of future research needs to be placed on characterizing the N metabolism and assimilation genes in the cultivated lettuce germplasm and introgress novel alleles from the wild species either by targeted approaches or by marker assisted breeding.

## Materials and methods

### Plant material and nitrogen stress treatment

Transcriptomes of cultivated iceberg lettuce (*L. sativa* cv. Salinas, hereafter referred to as Salinas) and its wild relative (*L. serriola* acc. US96UC23, hereafter referred to as UC) were explored in this study. Salinas is a shallow-rooted cultivar which was recently sequenced^85^ while UC is a deep-rooted genotype with greater tolerance to water stress and adverse conditions^86^. Thirty seeds of Salinas and UC were disinfected using 70% ethanol for 2 min and rinsed multiple time with autoclaved distilled water. Disinfected seeds were germinated on wet filter paper at 20 °C for 48 h and were transferred to 3.5 inches pots filled with sterilized potting mix consisting of vermiculite and perlite (3:1) for semi-hydroponic culture. Holding trays with pots were placed in a shallow tub with Hoagland nutrient solution (1 mM KH_2_PO_4_, 5 mM KNO_3_, 0.5 mM MgSO_4_.7H_2_O, 5 mM Ca(NO_3_)0.4H_2_O, 20 µM EDTA.Fe.Na, 25 µM H_3_BO_3_, 2 µM ZnSO_4_, 2 µM MnSO_4_, 0.5 µM CuSO_4_, 0.5 µM (NH_4_)_6_Mo_7_O_24_ (pH 6.5)). Seeds of the plant material for this study is provided by the USDA germplasm collection and were used with permission.This study comply with relevant institutional, national and international guidelines and legislation.

The plants were grown for two weeks in the growth chamber (CMP6050, Conviron, Winnipeg, Manitoba, Canada) at 20 °C under continuous white light (200 μmol m^-2^ s^-1^) and relative humidity between 50 and 70%. Nutrient solution was renewed every three days. Nitrogen stress (Low nitrogen; LN) was administered to two week old plants by transferring half of the plants to Hoagland nutrient solution with reduced nitrogen (0.5 mM KNO_3_ and 0.5 mM Ca(NO_3_)) and replacing appropriately with KCl and CaCl_2_ to equalize calcium and potassium concentration between treatments while the remaining constituents remained unchanged. The control plants (High nitrogen; HN) and nitrogen stress plants were grown for two more weeks and the respective nutrient solution changed every three days. Three whole healthy plants of each genotype from HN and LN group were sampled at four-week stage. All samples were washed, pat-dried and flash frozen in liquid nitrogen and stored at − 80 °C until RNA extraction.

### Phenotyping of nitrogen related traits

Chlorophyll fluorescence was measured using fluorescence imaging system PlantScreen™ (Transect FluorCam FC 800; Photon System Instruments, Brno, Czech Republic) with progressive scan CCD camera (effective resolution: 1360 × 1024 pixels) and a prime lens (Fujinin HF8XA-1). Plants were placed in a dark room for 30 min to open all PSII reaction centers. The kinetic chlorophyll fluorescence (ChlF) curves and images of the dark-adapted plants was acquired following Kautsky effect^87^ measured in pulse-amplitude modulated mode (PAM). Hyperspectral imaging was done using the visible near infra-red (VNIR) imaging unit on the PlantScreen™ consisting of a hyperspectral camera and a uniformly illuminating halogen lamp. The VNIR camera had CMOS sensors with 1920 × 1000 pixel resolution that can measure reflectance in spectrum from 350 to 950 nm. Vegetative Indices (VI) were calculated from the raw reflectance data using the hsdar package^88^ in RStudio. Chlorophyll content was measured on the youngest fully expanded leaf using handheld portable chlorophyll meter SPAD-502 (Soil Plant Analysis Development meter; Konica Minolta Inc, Osaka, Japan). Total nitrogen content was measured at the UC Davis Analytical Lab (http://anlab.ucdavis.edu/) from the leaves dried in oven at 65 °C for 72 h.

### RNA isolation and transcriptome sequencing

Total RNA was isolated from whole Salinas and UC plants grown under HN and LN treatments using Spectrum plant total RNA kit (Sigma-Aldrich, St. Louis, MO, USA). Preliminary quantitation of the RNA samples was done using NanoDrop (ThermoFisher, Waltham, MA, USA), RNA degradation and contamination was monitored on 1% agarose gels. RNA integrity of each sample was checked using Agilent 2100 bioanalyzer (Agilent Technologies, Santa Clara, CA, USA) at Novogene (Novogene, Sacramento, CA, USA) and sequencing was done using proprietary methods. Briefly, before library construction, total mRNA was enriched using enrichment oligo(dT) beads (New England BioLabs, Ipswich, MA, USA) and rRNA was removed by Ribo-Zero kit (Illumina, San Diego, CA, USA). cDNA libraries were constructed by randomly fragmenting mRNA, synthesizing second strand, and PCR enrichment. Concentration of libraries was checked by Qubit 2.0 (Life Technologies, Carlsbad, CA, USA) and insert size was checked using Agilent 2100 bioanalyzer. All the libraries were sequenced using 150 bp paired-end Illumina NovaSeq 6000 Sequencing System (Illumina, San Diego, CA, USA).

### Transcriptome assembly, and differential gene expression

Quality of raw data was checked by FastQC v0.11.3 (Babraham Institute, Cambridge, United Kingdom; https://www.bioinformatics.babraham.ac.uk/projects/fastqc/). Low-quality bases and adapter sequences from paired reads were trimmed using the Trimmomatic v0.30 program. The Q20, Q30, GC content, and sequence duplication level of the trimmed data were calculated. All the downstream analyses were based on the high-quality clean data. Clean reads were mapped to the *L. sativa* cv. Salinas reference genome downloaded from the comparative genomics research platform (https://genomevolution.org/coge/OrganismView.pl?oid=36218) using STAR aligner (v2.7.1a). Mapped reads were assigned to genomic features such as genes, exons and genomic windows by featureCounts program^89^ included in the subread v1.5.2 package (http://subread.sourceforge.net).

Differential gene expression analysis was performed using the R package DESeq2^90^. All calculated *P-values* were adjusted for multiple testing using the Benjamini–Hochberg procedure for a false discovery rate (FDR) of 5%. Libraries from LN treatments were compared to their respective controls (HN) to identify up and down regulated genes in each genotype. Genes were considered significant DEGs when their relative expression levels showed a 2-FC (log2 FC > 1.0 or < − 1.0) difference between LN and HN samples, with *P-value* < 0.05.

### Gene enrichment analysis

Enrichment analysis of the DEGs list was done by the gene list analysis tool PANTHER (http://www.pantherdb.org, Version 14.1, release date 12/03/2019) overrepresentation test using the Fisher's exact test with FDR multiple test correction (FDR < 0.05). Kyoto Encyclopedia of Genes and Genomes (KEGG) pathway enrichment analysis of the DEGs was performed using the Database for Annotation, Visualization and Integrated Discovery (DAVID) v6.8 (https://david.ncifcrf.gov) with Uniprot IDs associated with these genes GO terms with *P* < *0.05* were considered significantly enriched. MapMan analysis^91^ of the differentially expressing genes was performed to investigate major metabolic pathways affected by nitrogen stress.

### Gene co-expression network analysis

Gene clusters associated with the nitrogen metabolism were identified using the R package WGCNA (Langfelder and Hovarth, 2008) using the normalized count values of all the genotypes under LN and HN treatments. Network construction and module detection were performed using 1-step network construction and module detection function. Adjacency between the genes for constructing a weighted gene network was calculated by selecting appropriate thresholding power using ‘pickSoftThreshold’ function of WGCNA package (Fig. S1). Association between gene expression and nitrogen related phenotypic measurements were estimated using Gene Significance score. Furthermore, we calculated correlations of modules with gene expression profile (module members (MM)) and with traits (module eigengene (ME)) to evaluate significant relationships between the expression modules and genes with each phenotypic trait measurements. Two of the topmost correlated modules from WGCNA were further analyzed using topological centrality in R package Igraph^92^. We used betweenness centrality which is the number of shortest paths between every two other genes in the module and degree distribution of vertices which is the measure of the number of adjacent edges, for developing biologically informative networks. Network of the highly correlated genes were created using greedy algorithm (GLay) community clustering plugin^93^ of Cytoscape (version 3.8) software^94^ before visualization.

### Quantitative real-time PCR (RT-PCR) analysis

Eight nitrogen metabolism genes and three hub genes identified by WGCNA analysis were selected to validate RNA-seq results by RT-PCR using fast SYBR Green master mix (Promega, https://www.promega.com/). Gene specific primers designed using Primer3 version 0.4.0 (https://bioinfo.ut.ee/primer3-0.4.0/) following default parameters are shown in the Table S3. Two housekeeping genes, eukaryotic translation initiation factor 2 A (EIF2a; *Ls6_95581.1*) and isopentenyl diphosphate isomerase 2 (IPP2; *Ls2_17540.1*), were used as reference genes^95^. The relative expression values were calculated by the delta-delta CT method using the average of two reference genes and expressed as fold change referred to the expression of in the HN plants.

## Supplementary Information

Supplementary Information 1.

Supplementary Information 2.

Supplementary Information 3.

Supplementary Information 4.
