# Supplementary material for: Insights into nitrogen metabolism in the wild and cultivated lettuce as revealed by transcriptome and weighted gene co-expression network analysis
Source: Sci Rep. 2022 Jun 14;12:9852. doi: 10.1038/s41598-022-13954-z (PMC9197935; doi:10.1038/s41598-022-13954-z)
Supplement: Supplementary file 1 — Supplementary Information 1. [file 41598_2022_13954_MOESM1_ESM.docx]

Supplementary Fig. 1. Determination of the soft threshold in weighted genes co-expression network analysis. a. Analysis of the scale-free fit index for various soft-thresholds determining scale independence. b. Analysis of the mean connectivity for various soft-thresholds.

Supplementary Fig. 2. Scatter plots of module membership (MM) Vs gene significance (GS) in a. blue module and b. lightgreen module of different phenotypic traits in blue and light-green modules. GS represents the association between gene expression and phenotypic traits. In both modules, GS and MM have a high correlation.

Supplementary Fig. 3. Module eigengene adjacency heatmap. The heatmap shows the relatedness of the nine co-expression modules (color blocks on x-axis and y-axis) Relationship of the eight modules identified by WGCNA with the total nitrogen content. Red color represent high correlation and blue represent no correlation.

Supplementary Fig. 4. Class-wise counts of differentially expressing transcription factors (TFs) identified under nitrogen stress. a. up-regulated TFs, b. down-regulated TFs, c. total TFs

c

b

a
